# An Account of a Morbid Concretion Discharged from the Rectum of the Human Female, and in Its Chemical Characters Closely Resembling Ambergris; with Historical Remarks

**Published:** 1817-09

**Authors:** James Kennedy


### JOURNAL AND REVIEW.

VOL. IV.] SEPTEMBER, 1817. [NO. 21.

### PART I.

### ORIGINAL COMMUNICATIONS.

——— quid utile ———

### *An Account of a morbid Concretion discharged from the Rectum of the Human Female, and in its Chemical Characters closely resembling Ambergris; with Historical Remarks.*

By JAMES KENNEDY, M. D.*

INTESTINAL concretions have rarely fallen under the observation of medical practitioners. Cases, however, demonstrative of their existence, and accompanied by correct descriptions of their external characters, and of the phænomena which signalized their presence in the intestinal tube, are scattered in the records of pathological research. To this number an account of the present specimen is added, with no higher pretensions than those of a simple contribution, which may serve, in some degree, to elucidate the yet obscure history of these singular productions.

* I think it right to state, that the sketch of this paper was originally drawn up in the autumn of 1816; and at that time transmitted to my friend, Dr. Palmer, for publication. It has, however, been hitherto postponed for the purpose of revision; and thus has assumed the extended, and, I trust, more useful form in which it now appears. K.

C. D. aged 60, a widow, and mother of several children, has frequently, during the last two years, required my professional attentions: she is rather above the middle stature, of the muco-sanguineous temperament, regular in her habits, and inclined to corpulence. She has long been distressed by a morbid enlargement of the right inferior extremity. Twenty years ago, the limb, after suffering inflammation, became the seat of an ill-conditioned ulcer, which obstinately resisted every curative remedy. At length, however, unhealthy granulations sprang up, and cicatrization was completed; but the limb is yet often affected with an acute pain, of rheumatic character: occasionally, an acrid lymph exudes through the pores of the recently-formed cuticle and produces erosion, which is commonly soon remedied by the usual means.

The health of my patient was now sensibly impaired, and her constitution much weakened; her internal functions, particularly those of the stomach, were inertly and irregularly performed. Torpor of the intestinal canal prevailed. Alvine evacuation was difficult, painful, and defective. Hence the employment of laxatives was almost constantly, and that of purgatives and injections frequently required; and dyspepsia, fever, thirst, tormina, and tenesmus, undermined the strength, and destroyed the comfort of my patient. Her fortitude, however, and the natural cheerfulness of her disposition, opposed a powerful barrier to the encroachments of disease; and in fine weather she was enabled to walk about.

In May last, constipation of the bowels assumed an uncommonly obstinate form. For some days, my patient was much distressed by severe and frequent paroxysms of tenesmus. While straining violently in the act of evacuating the bowels, she felt an unnatural sensation within the orifice of the rectum; and hence was induced to make an examination with her fingers. A complete obstruction of the canal of the intestine filled her with alarm, and prompted her to request the assistance of her daughter. After reiterated attempts to dislodge the obstructing substance, during which pains more exquisite than those attendant on parturition were experienced, the concretion, which forms the subject of this communication was extracted. The bowels were now freely unloaded; the pain gradually gave way, and after two days, my patient resumed her former mode of life. She continues to regulate her bowels by the occasional use of compound rhubarb pill, and no new symptom of derangement in the internal functions has subsequently been developed.

### Description of the Concretion.

By the kindness of Dr. Andrew Ure, Professor of Natural and Experimental Philosophy and Chemistry in the Andersonian Institution of Glasgow, I am enabled to lay before the readers of this Journal a minute account of the external characters, and chemical analysis of the intestinal substance; some particulars respecting the symptoms and extraction of which I have just communicated. The "singular and interesting" results of this examination I cannot do better than state in the perspicuous language of Dr. Ure.

"The form of the alvine concretion, which you, some time ago, sent me for analysis, is that of a compressed cylinder; the length and large diameter, each one inch; the smaller, three quarters of an inch.* In hardness, it is equal to wax, but without its tenacity. One of the extremities, which is polished and glistening, exhibits the appearance of concentric laminæ formed by circular lines in a pale yellow basis: its sides have the lustre and marbled appearance of Castile soap. Its internal structure is granular, approaching to crystalline, with radiations from the centre to the circumference, of brown and bright yellow lines, possessed of pearly lustre. It is friable between the fingers, covering them, on pressure, with a mealy powder of but little unctuosity. Its weight is 167½ grains. The specific gravity of the mass at first seems inferior to that of distilled water: for the concretion floats upon it, but sinks, in a little while, to the bottom. In a solution of muriate of soda, sp. gravity 1.0135, a fragment of it remains suspended in any part of the fluid. This, therefore, is its specific gravity. Its odour is strong, but by no means disagreeable; it is decidedly musky, or, more precisely, that of ambergris."

"Water has no action on it; nor, when applied to moistened litmus paper, is the colour affected. It remains solid in boiling water; but, when raised to the temperature of about 400° of Fahrenheit's scale, fuses into a black mass, and exhales abundantly a white smoke, in the odour of which may be clearly traced that of ambergris, mixed

---

* When the concretion was first extracted, in May, 1816, it measured one inch and a half in length, and three inches and an eighth in circumference; and its weight was one hundred and eighty-four grains. The subsequent diminution of weight and volume, as specified by Dr. Ure, was owing to the removal of a portion of the substance, for experiment by myself. K.

with the smell of burning fat. Exposed in a platina capsule to a dull red heat, it burns with a vivid flame and much smoke, leaving no appreciable residuum. It dissolves rapidly in æther, forming an amber-coloured liquid. When the æther evaporates away, white glistening scales, of a micaceous appearance, are left. Ten parts of hot alcohol dissolve one part of it; but, as the alcohol cools, the greater part precipitates in these soft crystalline scales; while the surface of the liquid is covered with a beautiful iridescent pellicle, presenting stellated radiations. Naphtha, the fixed and volatile oils, readily act upon it, when they are slightly heated, forming bright yellow solutions. Small fragments of it, exposed on a sand-bath for two days, to the action of water of pure potash, in a glass capsule, were not altered in their volume or appearance. Neither does liquid ammonia, digested on it, produce the slightest effect.

"From the preceding remarkable analogies between the action of menstrua on this substance and on ambergris, I was led to imagine that the white smoke which it exhaled, when moderately heated, was benzoic acid. The alcoholic solution was, therefore, mixed with water of pure ammonia, when a milky liquid was produced from the separation of the substance in a pulverulent state from the alcohol. This mixture was evaporated by a gentle heat to dryness, in order to expel the uncombined ammonia and the alcohol. Warm water was then poured on the residuum, and the whole thrown on a filter. The liquid which passed through should contain benzoate of ammonia, provided any of this acid existed in the concretion: it was divided into two portions. Into one of these a few drops of dilute sulphuric acid were poured, and the acidulous liquid was then concentrated by evaporation in a glass capsule. On cooling, no traces of benzoic acid could be observed. An exceedingly minute quantity of benzoate of ammonia, treated in the same way, gave the characteristic crystals of that acid. The other portion was added to a neutral solution of the red muriate of iron; but it produced no turbidity. A very small particle of prepared benzoate of ammonia, added to the same muriate, speedily yielded a light brown precipitate; but produced no change whatever on the neutral solutions of the green sulphate or muriate of iron: a fact of some consequence as to the state of oxidizement in which iron exists in a mineral, or saline combination; leading also to an easy method of separating the two oxides from each other.

"Nitric acid, sp. gravity 1.300, digested on the concretion at a moderate heat, leaves it, on cooling, changed into bright yellow globules, denser and less friable than the original substance, and somewhat resembling resin. Their combustibility was unaltered.

"As this Institution possesses specimens of very fragrant ambergris, said to have been imported from Persia in their genuine state, I was anxious to compare the relations existing between them and this morbid concretion.

"Two of the pieces of ambergris differ considerably from each other. One is of a light grey colour, with resinous-looking points, and has a density somewhat greater than that of water. In this liquid, heated to 130°, it instantly falls down into bulky spongy masses. The other has a specific gravity of about 0.920, is of a darkish brown colour, and melts in water of the above temperature, into a viscid matter, of the consistence of treacle. Both are very soluble in warm alcohol, but the latter gives the richest golden-coloured solution: as the alcohol cools, a separation of brilliant scales takes place, and the liquid is covered with a silvery-looking pellicle. With æther, naphtha, and the fixed and volatile oils, the phænomena exhibited by ambergris, are absolutely the same with those which the concretion presents. The alcoholic solution, treated with ammonia, gives likewise the appearance of a milky emulsion. The effect of fusion in a capsule, placed over the flame of a lamp, is also the same; and a white smoke is exhaled, which, when the heat is considerably raised, becomes somewhat nauseous to the smell.

"In order to compare the relative effects of decomposition by heat still more exactly, three glass tubes, hermetically sealed at one extremity, were taken. Into the first I put a fragment of the concretion; into the second, a similar portion of the brown ambergris; and, into the third a few particles of the balsam of benzoin. Each fused at a gentle heat, with very similar appearances. The greater part of the ambergris rose to a considerable height in the tube, in the form of a brown oil: above which the white smoke condensed into crystalline needles. The same volatile oily-looking matter was also seen in the tube with the concretion, and a very little white pulverulent matter above it. On cutting the tubes across, the oil was found, in both cases, congealed into a tough resinous substance. In the third tube, crystals of benzoic acid were seen.

"The white crystalline matter from the ambergris powerfully reddened moistened litmus paper, and was evidently benzoic acid. But the whitish powder, coating the resin from the concretion, on being submitted to the same test, gave no satisfactory proof of an acidulous nature; and was in a quantity too minute for separate examination. We may hence infer, that the human concretion is probably destitute of the benzoic acid; which, therfore, still remains a chemical formation peculiar to the lower orders of animals. The substance in question is undoubtedly a species of ambergris.

"It differs very essentially from *adipocere*. This matter forms an emulsion with cold water; is fusible at the temperature of boiling water; unites with potash into a soap, with an evolution of ammonia; and gives a clear solution with liquid ammonia. The whole of the intestinal concretion is soluble in hot alcohol; while only one third of adipocere dissolves in that menstruum, at its boiling point. From ordinary *fat* it is easily distinguished by its solubility in æther and alcohol; its not combining with alkalies; and the high temperature required for its fusion. *Ambergris* and this morbid concretion are both formed in the same part of the body, namely, in the large intestines, perhaps in the rectum. The *physeter macrocephalus*, of Linnæus, is the species of whale which affords the ambergris. In the examination of Captain Coffin, before the Privy Council, in 1791, he stated, that "he found 362 ounces of ambergris in the intestines of a *female* whale, struck off the coast of Guinea. Part of it was voided from the rectum on cutting up the blubber; and the remainder was collected within the intestinal canal." The whales, which contain ambergris, are said "to be always lean and sickly, yield but very little oil, and seem almost torpid." Hence, when a spermaceti whale has this appearance, and does not emit fæces on being harpooned, the fishers generally expect to find ambergris within it. Whether this be the effect or cause of disease, is problematical; although the former seems to be the more rational conjecture. The curious fact, above stated, as to the sex of the whale, may lead us to speculate whether this production, now found also in the human subject, be peculiar to the *female* mammalia, and connected with lactation."

"In the second volume of *Dr. Monro's Outlines,** we have the analysis of some alvine concretions by Dr. Thos.

* Outlines of the Anatomy of the Human Body in its sound and diseased State. *Edinburgh*, 1813.

Thomson. The results obtained by this distinguished chemist shew, that the specimens which he examined were totally different from the concretion which I have now been analysing. ANDREW URE."

Anderson's Institution,
*Glasgow, June 12th*, 1817.

A few days after this communication came to hand, I was favoured by Dr. Ure with another, relative to the same interesting subject; which I shall correctly transcribe from the letter now before me.

"Since the details of my experiments on the alvine concretion were written, I have examined more particularly the ordinary ambergris; and conceive that the following facts may be worthy of publication.

"By adding alcohol to water, I formed a liquid of such density, at the temperature of 60°, that a fragment of the lighter specimen of ambergris, described in my last letter, remained at rest in any part of it. Its specific gravity was 0.959. The denser specimen was tried by suspension in water, in the usual way; and found to have a specific gravity of 1.200. The perfume of this is equally powerful and peculiar as that of the other specimen. A portion of the first being subjected to heat in a glass tube, over the flame of a lamp, fused very readily into a dark mass, and yielded much volatile oil, but no appearance of a crystalline acid; nor indeed could the slightest trace of acid be discovered by paper stained with litmus. I repeated this experiment with great care, and obtained the same result. The other specimen of ambergris, subjected to a similar treatment, fused less perfectly, but afforded, along with a volatile oil, a crystalline acid. This being dissolved in hot water, and added to neutral red muriate of iron, gave, after some time, a copious brown precipitate. As the smell of the volatile oil, exhaled in both cases, resembled very closely that of the oil of amber, I made some experiments, with a view of deciding whether the above acid were the benzoic or succinic. I do not know that any good criterion has hitherto been pointed out by chemical writers, whereby the point can be ascertained, with minute quantities of an impure acid. Both are volatile, crystallizable, and act similarly on red oxide of iron. I found it stated in that excellent body of chemical knowledge, *Aikin's Dictionary*, that succinate of ammonia sublimes without decomposition. Hence, I imagined that an easy mode might be devised of distinguishing the two acids, though in very minute quantities. I accordingly took some crystallized benzoate of ammonia, having a slight excess of acid, and subjected it to a moderate heat in a recurved glass tube, sealed hermetically at the lower extremity. The water of crystallization, which distilled over, was a concentrated solution of ammonia; and much pure ammoniacal gas was also exhaled. In the middle of the tube, pure benzoic acid was found in acicular crystals. I next took a few grains of the denser ambergris, digested it with alcohol, added water of ammonia to the solution, boiled, filtered, and evaporated to dryness. On re-dissolving, and slowly evaporating the solution, the saline matter obtained was in too minute a quantity to admit of the application of the above analytical criterion. Indeed, considering the very manifest portion of acid, which sublimes from a few grains of this ambergris, I expected a much more satisfactory portion of benzoate of ammonia. Is there some succinic acid formed during sublimation? This question I shall endeavour to resolve, when I have procured some new specimens of genuine ambergris: for I did not consider myself at liberty to use more than a few grains of that belonging to our Public Institution. From the whole of the preceding experiments, there seems to exist a perfect correspondence between the chemical properties of the human intestinal concretion, and the lighter variety of ambergris examined and described above. A. U."

*Glasgow, June 13th,* 1817.

---

The sketch of the case, which it was my object to record, thus being completed by the luminous and masterly statement of Dr. Ure, I have nothing more to add, nor do I consider necessary any comment upon it. I shall, therefore, conclude my paper, and perhaps not unacceptably to those readers who possess little leisure for extended literary research, with tracing a slight historical notice respecting intestinal concretions. It should, however, be understood, that I profess not to treat this part of my subject in a minute or comprehensive manner, but merely aim to collect within a narrow limit all the useful yet slender information connected with it, which may be gleaned from the library of a country practitioner.

*Bonetus** is the earliest writer in whose work I can find, clearly detailed, cases of concretions lodged in the intestinal canal. Two instances of this kind occur in the thir-

---

* *Sepulchretum sive Anatomia Practica,* &c.

teenth section of his third book. In the first, borrowed from *Ballonius,* a perforated concretion, affording passage to the liquid part of the fæces only, was found in the middle of the large intestine. Constipation, and the occasional discharge of liquid stools consequent on the use of purgatives, had been the chief symptoms during life.—The second case I shall almost literally translate: " A young person, without any other inconvenience, went rarely to stool. The excrements were dry, and (as though) calcined *(exusta)*: pains of the belly, with obstinate constipation, supervened. On *dissection,* a calculus, in size and figure resembling a chesnut, was found firmly adhering to the colon."*

The celebrated epistles of *Morgagni*† afford no very satisfactory information respecting these concretions. Some examples of them, which had fallen under the observation of *Bezoldus* and *Chomel,* are briefly noticed; but the illustrious Professor of Padua does not, himself, appear to have met with any. He supposes that many of the concretions, described as alvine, had really been formed in the gall-bladder, and acquired an increase of volume from deposition of successive strata in their passage through the intestines; and demonstrates the fallacy of the criterion whereby the pathologists of his time were wont to distinguish intestinal from cystic calculi.‡

*Lieutaud*§ records no instance of intestinal concretion as having been witnessed by himself. Several cases of stones *(Lapides)* in the stomach and intestines, borrowed from various sources, are inserted in his elaborate collection. As this work is probably in the hands of few of my readers, and withal rarely to be procured, I shall transcribe one or two of the cases from the sections in which they are respectively consigned.

*Stones in the Stomach.* || A man, aged 50, formerly a

---

* *Work just cited.* Tom. ii. p. 197.

† *De Sedibus et causis morborum, per anatomen indagatis, &c.*

‡ The inflammability of cystic calculi, and their possessing a specific gravity less than that of water, were properties incorrectly asserted by *Reverhorst* and others, constantly to distinguish them from the variety which is generated in the intestinal canal. " *Proba horum calculorum (biliariorum) est quod hi, igni admoto, flammam nontantùm concipiant, sed et in aquam projecti, minimè fundum petant, sed eidem supernatent propter bilis particulas oleosas, hos lapillos componentes.*" Libr. iii. Epist. xxxvii. Art. 25.

§ *Historia Anatomico-Medica, &c.*

|| *Work just cited.* Tom. i. p. 17.

soldier, was attacked with pain of the stomach, exceedingly severe, and aggravated on taking food, so that for two years preceding his death, he lived on wine alone.—On *dissection*, the stomach was found diminished in volume and corrugated; and in it a very hard egg-shaped, and somewhat rough stone, of an ash colour, and weighing four ounces.—A man, of 90 years, who, during his whole life, had drank cold spring water every morning, suffered, for a few months before his decease, a violent and irremediable pain of the stomach. In this organ, a calculus, as large as a filbert, was found after death. The other viscera were sound."

*Stones in the Intestines.** "A thin young man, subject to costiveness, experienced dreadful pain of the bowels in going to stool. Some ash-coloured stones, varying from the size of a vetch to that of a filbert, were occasionally voided. The pains increased, and he died from obstructed bowels. On *dissection*, a calculus, of the size and figure of a chesnut, was found firmly lodged in the colon."—A case from *Severinus* is related, wherein the colon of a man, destroyed by "colic pains," contained "a stone of the form and volume of a goose egg: and one, from *Schneiderus*, of a blacksmith who had suffered from obtuse gastric pain; and whose jejunum, on *dissection*, was found completely obstructed by a large whitish stone."

*Marechal*† has recorded the case of a lady, in whom the exhibition of a glyster was prevented by the lodgment of a large calculus in the superior part of the rectum. After having duly dilated the anus, this celebrated surgeon extracted the stone. It weighed two ounces and a half, and was of an ovoid form. Its greater diameter was two inches eight lines; its smaller, one inch seven lines. When laid on hot coals, part of it deliquesced: the remnant gave out flame and was calcined. Hence it was supposed to consist partly of bilious, and partly of stercoral matter.

The venerable *Portal*,‡ in the course of a long and extensive practice, and amid the numerous dissections which his academical lectures must have required him to perform, seems very rarely to have observed these concretions. His observations relate both to the gastric and intestinal variety; and, as they are not very prolix, I shall proceed to transcribe them. Speaking of the former kind

---

* *Work just cited*, p. 78.

† *Memoires de l'Academie Royale de Chirurgie.*

‡ *Cours d'Anatomie Médicale*, &c. Paris, 1803.

of concretion, the Professor says, "I found in the stomach of a subject, opened for one of my anatomical lectures, a concretion of the form and volume of a pigeon's egg. In its centre were contained two or three other concretions, which crackled in the fire. Its substance was yellow and bitter, really bilious. In some stomachs, concretions have been found, possessing the solidity and figure of urinary calculi."* Gastric concretions may be loose in the cavity, adherent to the parietes, or encysted between the membranes, of the stomach. He adds, that *Ruysch* preserved in his Museum, "a tumour found in the stomach, and containing molar teeth and hair." With respect to intestinal accumulations, he observes, "Calculous concretions sometimes form in, and obstruct, the intestinal canal. Alimentary or fæcal matter accumulates above; its progress is interrupted; obstinate constipation succeeds, with dreadful vomiting, and frequently ushered in by violent colic and inflammatory symptoms. Such concretions may be either altogether bilious, or formed of a nucleus of bile, upon which are deposited strata of other substances, alimentary, fæcal, lymphatic, or viscous. Calculi, as large as a pullet's egg, and possessing a different structure, have also been found in the intestines. I have seen some which had for their nucleus a concretion, of the nature of pancreatic calculi.†

By *Dr. Parre,*‡ the Dictionary-writer, nothing worthy of particular notice is mentioned respecting intestinal calculi. He contents himself with a cursory allusion to cases of the kind, recorded by *Molinelli, Gooch, Houstan,* and *Horstius.*

Our own celebrated Pathologist, *Dr. Baillie,*§ acknowledges, that accumulations of calculous matter in the intestinal canal, have never fallen under his observation; and states his belief that, in the human subject at least, they are of "very rare occurrence."

The last author, whose labours it remains for me to notice, in concluding this hasty retrospect, is *Dr. Monro,*‖ the present Professor of Medicine in the University of Edinburgh: and the account delivered by him, of alvine

---

* *Work just cited,* tom. v. p. 192.

† *Work just cited,* tom. v. p. 239.

‡ *London Medical Dictionary, Appendix.* Article, *Calculus.*

§ *Morbid Anatomy,* &c. edit. of 1807, p. 195-196.

‖ *The Morbid Anatomy of the Human Gullet, Stomach, and Intestines.* Edinburgh, 1811.

concretions, is by far more clear and comprehensive than any which I have elsewhere read. Indeed, it forms a nearly complete, though brief, medical history of these morbid productions. The Professor's work, however, being too expensive for very general perusal, I shall perhaps be conferring a benefit on those who possess not the valuable original, by presenting a slight analytical sketch of what Dr. Monro has written upon the subject.

Forty-two specimens of alvine concretion, collected by the present Professor's father, are preserved in the Museum of the University of Edinburgh. I shall briefly retrace, in their respective order, the observations of the Doctor, with regard to the external and physical characters, chemical analysis, effects, symptoms, and treatment, of this singular class of morbid substances.

*External and physical characters. External colour*, smaller concretions destitute of coating, and resembling bad yellow ochre; larger, encrusted with an earthy matter, of a coffee colour, and purple, or sometimes white. *Size*, generally varying from a pea to that of an orange; sometimes such as to weigh four pounds. *Figure*, in the larger, very irregular, from the inequalities of the external crust; sometimes formed of a congeries of smaller calculi; sometimes perforated like coral: in the smaller, oval or spherical, somewhat flattened at the sides; sometimes so deeply impressed, one by the other, as to resemble cup and ball. *Internal structure*, porous, like dried sponge; seen, under a magnifier, to be composed of numerous minute fibres, intimately interwoven like those in hat or chamois leather; the interstices filled with earthy matter; may be cut with a sharp instrument, but crumble before a blunt one; cavities sometimes penetrating to the centre, and filled with viscid mucus: the external crust sometimes only partial, rarely more than two or three lines thick; stratified; friable, and apt to crack off when dry: the more internal substance sometimes composed of distinct layers of yellow or brown, determined in their course by the external figure of the concretion, and pervading every part of it, or imperfect; not forming one continued line as in biliary or urinary calculi: sometimes no strata observed. The nucleus commonly a cherry-stone, small piece of bone, or biliary calculus. Other varieties have been seen composed exclusively of magnesia, which had been inordinately taken during life; or precisely resembling biliary concretions; or consisting principally of inspissated lymph, by which cherry-stones, or other extraneous substances, are bound together. *Number,* rarely more than two; but sometimes very considerable.*

*Chemical analysis.* The following are the results obtained by Dr. Thomas Thomson, from an examination of the specimens contained in the Edinburgh Museum. His name will be regarded as a sufficient pledge for their correctness. The concretions, at first, swam in water, but soon permanently sank. Specific gravity of different specimens varying from 1.376 to 1.540: average, about 1.400. Cold water acquired from them a brownish tinge, and took up albumen, separated in white flakes by boiling: there were also a peculiar brown substance, at first dissolving in water, but rendered nearly insoluble by slow evaporation; soluble in alcohol; most nearly resembling vegetable extractive, but too small in quantity for exact examination; muriate of soda, crystallizing on spontaneous evaporation of the water; phosphate of lime, precipitated by ammonia; sulphate of soda in a minute proportion; and, perhaps, sulphate of lime. Alcohol dissolved the peculiar brown matter, and some of the salts; caustic pot-ash, the albumen, brown matter, and, perhaps, some of the salts; and muriatic acid, a proportion of phosphate of lime. After all, there remained a peculiar substance, having the colour and texture of the calculus;† in very short threads, light, resembling cork, or rather the fungus called *amadou*‡ by the French; tasteless; insoluble in water, alcohol, æther, pot-ash ley, and muriatic acid; blackening, and partly reduced to charcoal by sulphuric acid; slowly dissolving by heat, without effervescence, in nitric acid, and leaving, on evaporation to dryness, a whitish residue, of bitter taste, and imperfectly soluble in water; not converted, by repeated digestion with this acid, into any of

---

* The following is a description of one of the more common specimens of alvine concretion, drawn up by Professor Jameison, after the manner of Werner: "*Colour,* of the external surface, dark ochre yellow. The cut surface exhibits concentric stripes of different breadths. The broadest stripes are nearly olive green; the narrower, yellowish grey and ash grey. *Shape,* botryoidal; in some places inclining to tuberose. *Lustre,* dull. *Fracture,* of the broad stripes, very delicate, promiscuous, fibrous; of the narrow, apparently even. *Transparency,* opaque. *Streak,* unchanged in the streak. *Tenacity,* sectile. *Frangibility,* difficultly frangible. *Feel,* feels fine and rather meagre." *Morbid Anatomy, &c.* p. 36-37.

† Ten grains of the calculus left 1.2 grains of this matter.

‡ Agaric of the oak — "*l'agaric de chêne ou l'amadou.*" *Dict. des Sciences Méd.* Art. *Agaric.* K.

the vegetable acids; burning with slight flame, rather like a vegetable than animal body, but differing from all animal and vegetable substances hitherto examined, and distinguishable from wood by its insolubility in pot-ash ley. The calculi consisted of alternate layers, or intimate mixture, of this substance and phosphate of lime, to which the albumen and brown matter served as a cement, the other substances being in small proportions. Phosphate of lime, mixed with a brown animal matter, formed the external crust of some of the specimens: on the surface of a few, crystals of phosphate of ammonia and magnesia were observed. The presence of neither pot-ash, ammonia, carbonate of lime, uric acid, nor urea, could be detected. Dr. Thomson concludes this statement by observing, "that the calculi in question are of a very different nature from any hitherto examined. They are of a much more insoluble nature than urinary calculi: indeed, no solvent exists, except of a nature so corrosive that it could not be applied."*

*Effects of concretions on the intestinal canal.* These effects are, irritation, and sometimes active inflammation, of the intestines, from mechanical obstruction of the fæces; lodgment of the extraneous body in a sac formed by the coats of the intestines, and unnatural distension of the alvine tube immediately above; and stricture of the intestines. Sometimes the concretion adheres closely to the mucous membrane.

*Symptoms.* Griping and acute pains in the bowels, sometimes fixed, commonly aggravated by the use of acids and indigestible food, and frequently accompanied by nausea and vomiting: sometimes costiveness, with constant inclination to go to stool; sometimes watery dejections, with discharge of viscid mucus or blood, productive of great relief; sometimes involuntary stools; indigestion, weakness, extreme emaciation: pulse in the commencement of the disease little affected; a hard, painful, globular tumour, generally perceptible on relaxation of the abdominal parietes, most frequently in the course of the large intestines; rarely changing, although often appearing to change, its place, especially when it is lodged within the small intestines or arch of the colon, from the latitude of motion allowed to these viscera by the mesen-

* *Morbid Anatomy*, p. 44-48. This is the analysis alluded to by Dr. Ure in the preceding pages. K.

tery and mesocolon. Great pain in the pelvis, with apprehension of instant death, obstruction of the bowels, and sometimes retention of urine, are caused by descent of the calculus into the rectum, from whence it may be discharged spontaneously with the fæces, or extracted by the fingers or surgical instruments, as in the cases described by myself and Marechal, and in three others, mentioned by Dr. Monro, as having been communicated to his father.*

*Diagnosis* of the presence of alvine concretions from other abdominal lesions is not attempted by the Professor. This is to be regretted; as there are few so well qualified as himself, by experience and reflection, to fill up the void in the history of this rare disease. Sensible of my own incompetence for the task, I shall decline it. Tumours, situated in any portion of the intestinal tube, I may, however, be allowed to observe, and particularly scirrhi of the pylorus and colon, appear, theoretically viewed, to be the forms of abdominal malady, which might be most readily mistaken for the presence of these morbid concretions.

With respect to *Treatment*, Dr. Monro recommends, that, in the earlier stages of the complaint, while the concretion remains moveable within the intestine, attempts should be made, both by mechanical means and lubrication of the canal with proper remedies, to effect the descent of the extraneous substance into the rectum, whence it may be naturally discharged or artificially extracted. With this view, Dr. Monro, senior, directed laxatives, emollient glysters; occasional purgatives, followed, at the commencement of their operation, by an emetic and warm bath; the internal use of Castile soap and oil, and frequent attempts by the patient, especially when at stool, to work the calculus downward with his hand, assisted by an effort to vomit, artificially induced; and light and simple diet. But these means do not seem to have been effectual. Two cases, most interestingly related, wherein they were tried by the Doctor's prescription, terminated fatally. When, however, the concretion is of long standing, and, from having become lodged within a sac † of the

* See *Morbid Anatomy*, &c. p. 50-51.

† A concretion may commonly, at first, be moved from one part of the intestinal canal to another; and, if there be two or more, they may be made to strike against each other. But they afterwards become immoveable; and this circumstance, in the opinion of Dr. Monro, "merits the colon, is no more moveable, Dr. Monro proposes its extraction from the intestine, by the hand of the surgeon, as the only mean whereby the life of the patient can be preserved; and transcribes the directions laid down, in a similar case, by his father, for this novel and daring operation.* The experienced surgeon would, however, I think, feel no common reluctance in staking his character upon an enterprize, so obviously perilous and difficult in execution, and uncertain in result: nor can I, perhaps, more effectually repress the ardour for dashing experiment in the young and sanguine, than by repeating the timely and judicious admonition of a critical writer in a valuable contemporary Journal. "Upon the very bold operation of cutting out these concretions, when lodged in the colon, proposed by Dr. Monro, senior, and recommended by our author, we think it our duty to state, that the diagnosis is so difficult, that in one case, where the operation was strongly advised, it turned out, upon dissection, that the disease was a *scirrhous pylorus*."†

Such is the brief historical sketch on the subject of intestinal concretions, which my limited acquaintance with medical literature has enabled me to introduce here: for, whatever of originality it may possess, I consider myself wholly indebted to the kindness and talents of Dr. Ure. The more humble merit of diligent research and compilation is all I arrogate to myself. The fact of a morbid substance, closely resembling ambergris, having been generated within the intestines of the human subject, although in itself exceedingly interesting and curious, leads not directly to any practical inference or improvement. To make novelty the vehicle of usefulness, at least to the junior members of the profession, has therefore been my aim; conscious that the writer, who has little of his own to communicate, may often essentially serve the cause of science, by concentrating within a narrow and readily accessible space the labours of other men; and deeply convinced, that the researches of the pathologist are only really valuable, as they throw light upon the road of practice, and thus contribute to prolong the existence, and ameliorate the condition, of the human race.

*Dunning, Perthshire, July* 1817.

---

most particular attention, as it marks the progress of the disease, and points out the chance there may be of dislodging the concretion. *Morbid Anatomy*, &c. p. 52.

* See these directions, *Morbid Anatomy*, &c. p. 63-64. K.

† *Edinburgh Medical and Surgical Journal*, No. xxxiii. p. 112.

